# Novel methodology for pharmaceutical expenditure forecast

**DOI:** 10.3402/jmahp.v2.24082

**Published:** 2014-11-27

**Authors:** Anne-Lise Vataire, Laurent Cetinsoy, Samuel Aballéa, Cécile Rémuzat, Duccio Urbinati, Åsa Kornfeld, Olfa Mzoughi, Mondher Toumi

**Affiliations:** 1 Creativ-Ceutical, Paris, France; 2 Creativ-Ceutical, Milano, Italy; 3 University of Marseille, Marseille, France

**Keywords:** forecast model, pharmaceutical expenditure, health policy, generic, biosimilar, innovative medicine

## Abstract

**Background and objective:**

The value appreciation of new drugs across countries today features a disruption that is making the historical data that are used for forecasting pharmaceutical expenditure poorly reliable. Forecasting methods rarely addressed uncertainty. The objective of this project was to propose a methodology to perform pharmaceutical expenditure forecasting that integrates expected policy changes and uncertainty (developed for the European Commission as the ‘EU Pharmaceutical expenditure forecast’; see http://ec.europa.eu/health/healthcare/key_documents/index_en.htm).

**Methods:**

1) Identification of all pharmaceuticals going off-patent and new branded medicinal products over a 5-year forecasting period in seven European Union (EU) Member States. 2) Development of a model to estimate direct and indirect impacts (based on health policies and clinical experts) on savings of generics and biosimilars. Inputs were originator sales value, patent expiry date, time to launch after marketing authorization, price discount, penetration rate, time to peak sales, and impact on brand price. 3) Development of a model for new drugs, which estimated sales progression in a competitive environment. Clinical expected benefits as well as commercial potential were assessed for each product by clinical experts. Inputs were development phase, marketing authorization dates, orphan condition, market size, and competitors. 4) Separate analysis of the budget impact of products going off-patent and new drugs according to several perspectives, distribution chains, and outcomes. 5) Addressing uncertainty surrounding estimations via deterministic and probabilistic sensitivity analysis.

**Results:**

This methodology has proven to be effective by 1) identifying the main parameters impacting the variations in pharmaceutical expenditure forecasting across countries: generics discounts and penetration, brand price after patent loss, reimbursement rate, the penetration of biosimilars and discount price, distribution chains, and the time to reach peak sales for new drugs; 2) estimating the statistical distribution of the budget impact; and 3) testing different pricing and reimbursement policy decisions on health expenditures.

**Conclusions:**

This methodology was independent of historical data and appeared to be highly flexible and adapted to test robustness and provide probabilistic analysis to support policy decision making.

With the economic crisis of 2008 and the substantial increase in public budget deficits, governments have implemented austerity plans to lower debt levels. The ever-growing pharmaceutical expenditure became a major target of healthcare cost-containment efforts, and several measures were implemented in European countries to contain public medicine expenditure. Common measures included price reductions; changes in the co-payments, in the Value-Added Tax rates on medicines, and in the distribution margins; as well as generics and biosimilars promotion (1, 2). National authorities have increased their use of health technology assessments (HTA) authorities to assess the impact of a new technology. These authorities became a focus for Europe with the establishment of the European Network for HTA (EUnetHTA) in 2005 (2, 3). Today, decisions regarding pharmaceutical products appear stricter than in previous years with a growing aversion to uncertainty from HTA agencies and payers (4, 5). These policy changes created a disruption in pharmaceutical market access and prices, making the historical data that are used for forecasting pharmaceutical expenditure poorly reliable because they do not meet new pricing and market access practices.

A review of the main existing models related to pharmaceutical expenditure forecasting showed an increase in health expenditures over the years. Indeed, using a Markov micro-simulation model based on a French patient database to measure the impact of ageing and chronic conditions on the evolution of future drugs expenditure from 2004 to 2029, Thiébaut et al. found that reimbursable drug expenditures will increase between 1.1 and 1.8% per year due to epidemiological and life expectancy changes (6). Connor et al. and Keehan et al. forecasted an increase in health expenditure over the next year (7, 8). Connor et al. (2003) used a mix of statistical analyses of prescription database (IMS) and expert opinion to generate forecasting based on historical trends and the potential market. A similar methodology was also used by Keehan et al. in 2011 for their United States (US) study. Both studies forecasted an increase in health expenditure over the next year (7, 8). Their prediction was based on the GDP and the insured number of persons’ evolution. Both studies forecasted an increase of health spending over the coming years. Furthermore, Wettermark et al. showed an increase of 2.0% in total expenditure for prescription and hospital drugs in 2010 and of 4.0% in 2011, using a linear regression analysis on historical IMS aggregate sales data between 2006 and 2009 to predict future expenditure for 2011–2012 (9).

Although these models allowed expenditure forecasting, they rarely addressed uncertainty and are therefore inappropriate in a fast-changing policy environment with difficult prediction of future policy landscape. This review of models also showed that there were no publications modeling the whole process of savings due to products going off-patent (biosimilar and generic medicinal products) and additional costs of new branded medicinal products at drug level.

The objective of this project was to develop a methodology to forecast pharmaceutical expenditure while integrating expected policy changes and uncertainty in seven European Union (EU) Member States (MS) (France, Germany, Greece, Hungary, Poland, Portugal, and the United Kingdom (UK)) over a 5-year forecasted period (2012–2016). This project was performed for the European Commission (‘EU Pharmaceutical expenditure forecast’; see http://ec.europa.eu/health/healthcare/key_documents/index_en.htm).

## Method

**Fig. 1:**
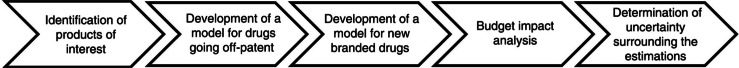
Five-step methodology for pharmaceutical expenditure forecast.

**Fig. 2:**
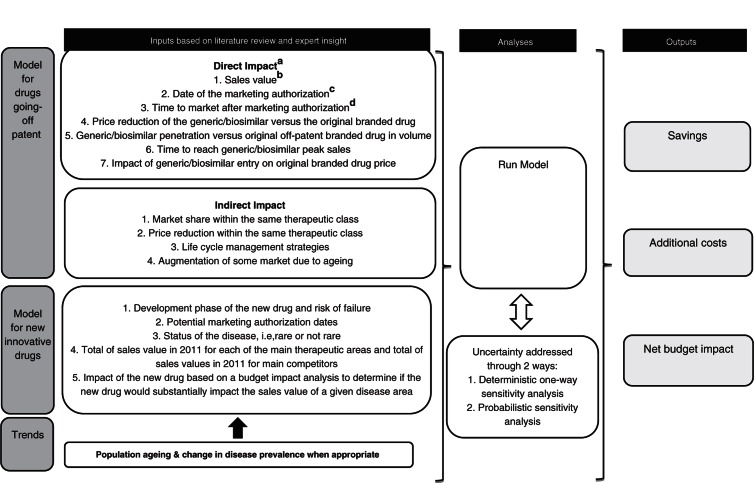
Model concept. ^a^The direct impact on savings corresponded to the value extrapolated from the market share evolution of the generic or biosimilar drug versus the original branded drug. ^b^Sales value for 2011 of the original branded drug extracted from the IMS database, including hospital and retail sales as well as manufacturer and public sales. ^c^Date of the marketing authorization, which is assumed to be the same as the date of patent expiry of the original branded drug. Indeed, with the provisions of the EU regulatory system, it is not allowable to link marketing authorization to the patent status of the originator reference product (12). ^d^For generics and biosimilars distributed through the retail chain, the time to market after marketing authorization corresponded to the delays of pricing and reimbursement status after marketing authorization. For biosimilars, time to uptake was also considered for the time to market. Indeed, low biosimilar uptakes are reported in Europe due to the reluctance of physicians to prescribe them and due to the fact that, unlike generics, they cannot be substituted. Generics and biosimilars distributed through the hospital chain were assumed to reach the market after marketing authorization without any delay.

The methodology for pharmaceutical expenditure forecast included five main steps (Fig. 1) and involved a board of six experts with a strong experience in market access of healthcare products and healthcare policies. The study board of experts acknowledged the need for country-specific qualified advisors that were identified and provided inputs at various stages of the project. Therefore, the MS’ specificities were taken into account as far as possible in the forecasting model. The model concept is illustrated in Fig. 2.

### Step 1: Identification of products of interest

Products going off-patent (biosimilar and generic medicinal products) and new branded medicinal products in the selected EU MS were identified for the period 2010–2016. Only products that went off-patent or were approved in 2010 and 2011 were expected to impact the pharmaceutical budget on the forecasted period 2012–2016.

Several sources of information were used to get an exhaustive list of products going off-patent and all new branded medicinal products as described below.

Products going off-patent were identified through several sources of information, such as Datamonitor reports, the PharmaVitae database, the Medtrack database, patent databases (free access patent databases such as the World Intellectual Property Organization, the European Patent Office, and Espacenet; and commercial online databases, such as STN International/CAPADOC, Questel Orbit, and Genericsweb), pharmaceutical company websites, press releases, investors’ reports, and the IMS database, as well as an in-house proprietary drug information database. For biosimilar drugs, exact patent expiration dates are generally hard to establish because of the lack of centralized listing of biological patents and the fact that expiration dates are proprietary information to the companies. Moreover, each product has formulation patents that are difficult to assess. Biological expirations over the forecasted period were therefore mainly extracted from the Generics and Biosimilars Initiative (GaBi) (10).

New branded medicinal products were identified by cross-checking several databases such as European Medicines Agency (EMA) public information, Datamonitor reports, the PharmaVitae database, the Medtrack database, pharmaceutical company websites and press releases, investors’ reports, an in-house proprietary drug information database, and study registries such as clinicaltrials.gov.

New drugs were selected according to the following criteria:

1. Drugs that could be approved or were approved via the EMA procedure, that is, drugs for the treatment of Human Immunodeficiency Virus, cancer, diabetes, neurodegenerative diseases, immune dysfunctions, and viral diseases; medicines derived from biotechnology processes; advanced-therapy medicines, orphan medicines; or medicine considered as a significant innovation or of interest of public health (11).
2. Only the first approval of new entities was considered. Renewals, variations, or updates were excluded.
3. Only products that had a positive phase IIb on the primary endpoint were taken into account, in order to minimize uncertainty and be consistent with the time window considered for this project. Orphan drugs with an ongoing phase II, which due to the orphan procedures could potentially reach the market during the forecasted period, were considered as being potential new drugs.
4. In light of the development time required for new vaccines (i.e., delays in coverage for new vaccines are considerably longer than those for other pharmaceutical products), vaccines were not considered to have a significant budget impact in the 5-year period of interest.

### Step 2: Development of a model for drugs going off-patent

This model estimated a separate and combined effect of the direct and indirect impacts on savings from the genericization of the market for each year in the forecasted period based on the literature review, the local expert consultation, and the study board of experts’ insight. Inputs considered to assess the direct and indirect impacts of generic entry are presented in Fig. 2.

For this model, the sales evolution of generic and biosimilar products was assumed to be linear for the retail chain distribution until reaching peak sales, considering that the largest budget impact would happen after peak sales. Once the peak of sales was reached, it was assumed that sales would remain stable over time. For the hospital distribution chain, peak sales were assumed to be reached at day 1, as hospitals optimize their purchaser position through tenders.

The direct impact on savings corresponded to the value extrapolated from the market share evolution of the generic or biosimilar drug versus the original branded drug.

Seven inputs were considered to calculate the direct impact:

1. Sales value for 2011 of the original branded drug extracted from the IMS database, including hospital and retail sales as well as manufacturer and public sales.
2. Date of the marketing authorization, assumed to be the same as the date of patent expiry of the original branded drug. Indeed, with the provisions of the EU regulatory system, it is not allowed to link marketing authorization to the patent status of the originator reference product (12).
3. Time to market after marketing authorization.
  - For generics and biosimilars distributed through the retail chain, the time to market after marketing authorization corresponded to the time delays of pricing and reimbursement status after marketing authorization. For biosimilars, time to uptake was also considered for the time to market. Indeed, low biosimilar uptakes are reported in Europe due to the reluctance of physicians to prescribe them and due to the fact that, unlike generics, they cannot be substituted.
  - Generics and biosimilars distributed through the hospital chain were assumed to reach the market after marketing authorization without any delay.
4. Price reduction of the generic or biosimilar drug versus the original branded drug.
5. Generic or biosimilar drug penetration versus original off-patent branded drug in volume.
6. Time to reach generic or biosimilar drug peak sales.
7. Impact of generic or biosimilar drug entry on original branded drug price.

The assessment of the indirect impact of generic entry included:

1. The market share within the same therapeutic class.
2. The price reduction within the same therapeutic class.
3. The life cycle management strategies.
4. The augmentation of some market due to ageing.

### Step 3: Development of a model for new innovative drugs

The model estimated the value of sales and progression of market share of new innovative medicines in a competitive environment and took into account the risk of failure regarding the development of the drug per therapeutic class using the results from a study conducted by DiMasi et al. (13).

New drugs were looked at individually to assess their clinical potential and translate it into commercial potential. Five inputs were considered to calculate the potential impact of the new approved medicines (Fig. 2):

1. Development phase of the new drug.
2. Potential marketing authorization dates.

Potential marketing authorization dates were based on Datamonitor reports, the PharmaVitae database, the Medtrack database, pharmaceutical companies websites, press releases, investors’ reports, and clinical trial registries such as clinicaltrials.gov. Dates were reviewed and validated by the study board of experts based on the available clinical trials performed, the initiation date of ongoing phase III trials, recruitment progress, the disease area, and the date of marketing authorization application filing to the EMA, if applicable. These rules were modulated based on the number of patients to be recruited, the evidence of the recruitment speed based on clinical trial registries when well updated, the trial sample size, and the disease area, as the speed of the running trial is not consistent across diseases.

- Status of the disease (i.e., rare or not rare)
- Total sales value in 2011 for each of the main therapeutic areas and total sales value in 2011 for the main competitors

The sales of the new drug, in all countries with the exception of Germany, were assumed to impact the market 1 year after the date of marketing approval in the EU due to pricing and reimbursement policies. For Germany, the impact was assumed to be at market approval, as companies initiate sales based on a free pricing for the first year following the launch of new innovative drugs.

1. In the case of products used to treat rare diseases, peak sales were considered to be achieved over a year, due to the important unmet needs for therapies in these disease areas, the lack of alternative therapies in most cases, and the targeted prescribers who are well informed of any new treatment options. For other products, sales were considered to reach peak over a period of 3 years.
2. Impact of the new drug based on a budget impact analysis to determine if the new drug would substantially impact the sales value of a given disease area.

The study board of experts reviewed evidence on either efficacy or safety improvements provided by the new drug over the available drugs in the same therapeutic area to determine if the new drug could have a significant budgetary impact. The board of experts took into account new attributes brought by the product from the perspective of the HTA in place for each country. A new drug should have added clinical value to be considered as impacting the sales of a therapeutic area. In principle, this meant that the generated sales of products with similar clinical value would only replace sales of existing products. This was consistent with actual regulations within EU countries. When assessing the potential clinical benefit of the new drugs on the forecasting period, the study board of experts considered the increase in pressure on pharmaceuticals to evidence additional clinical or economic benefits to achieve market access.

French Health Authority (HAS) assessments were used whenever available for already approved drugs (2010–2011), and the budget impact was considered only for new medicines with the following Improvement of Medical Benefit (Amélioration du Service Médical Rendu, or ASMR) scores rated on a 5-level scale: ASMR I (major improvement), II (important improvement), and III (moderate improvement). Medicines with ASMR IV (minor improvement) and V (no improvement) were not considered to have an impact on budget. The HAS clinical perspective was complemented by Scottish Medicines Consortium (SMC) reviews to provide a perspective on the incremental cost-effectiveness.

For new drugs that were not approved yet, clinical results of the phase II study were reviewed through related press releases, company websites, trial registries, conference abstracts, and analyst reports and used to assess the potential clinical benefit.

The following new drugs were assumed not to have any budget impact: combinations of already approved products, unless there is a major added value in terms of efficacy or safety versus the combination of the active compounds taken separately; me-too drugs; and new formulations of approved products.

Specific assumptions were adopted for the oncology area, and impact assessments were based on the overall survival. Products were considered as not assessable when the overall survival benefit was not available. The impact was then quantified by the study board of experts, case by case, based on the current prices of existing alternative treatments, the magnitude of overall survival, the severity of the condition, the existence of effective therapies, the market share of different products, and any relevant information specific to each case. New oncology drugs, as a result of their costliness, were assumed not to have an important development in lower income countries (Poland, Hungary, Greece, and Portugal). A different methodology was implemented to estimate the penetration rate's evolution in these countries. Oncology drugs sales’ proportions were estimated in lower income countries with French sales in 2011 as a reference. The estimated proportion was considered as constant during the forecasted period.

### Step 4: Budget impact analysis

The budget impact of products going off-patent and the impact of new branded drugs were analyzed separately according to three perspectives:

1. The manufacturer's perspective, by taking into account manufacturer ex-factory sales.
2. Society’s perspective, by integrating all sales based on retail and hospital sales irrespective of reimbursement.
3. The Healthcare public payer’s perspective, by applying the reimbursement rates in place in each EU MS to public sales.

**Fig. 3:**
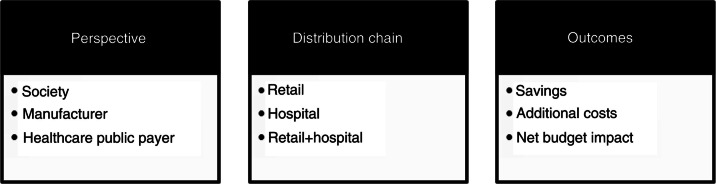
Description of model characteristics.

These budget impacts were also analyzed separately according to the following (Fig. 3):

1. One global rate for reimbursement rates: the decision was made to apply one global rate per country on total pharmaceutical expenditures based on the mean of co-payment levels in place in each country. This assumption appeared to be quite reasonable considering that the main pharmaceutical classes were represented in our model. As such, applying different levels of reimbursement rates was expected to lead to similar results as applying an average rate across the different therapeutic areas. Furthermore, reimbursement rates also depend on patient diseases, patient ages, the cost of medicine, and so on in most countries. We assumed that applying this reimbursement rate for all consumed medicine, including in hospitals, would not substantially affect the results of our model.
  - Several types of distribution chain (retail, hospital, combined retail, and hospital)
  - Several outcomes (savings due to products going off-patent, additional costs due to new innovative products, and net budget impact)^1^

Ageing was taken into account directly in the input parameters of the model and indirectly for prevalence calculation purposes, using the evolution of the population structure (the average evolution from 2012 to 2016 derived from the 2012 ageing report) (14). These data were expected to be very robust as population ageing anticipation for the next 5 years is a very well-defined process with very little uncertainty. Thus, based on the high level of certainty of ageing over a 5-year period, ageing was not implemented in the sensitivity analysis.

Prevalence data were mainly extracted from Datamonitor epidemiology reports. When these were not available, various public sources were searched, including Medline and Embase and web sources (especially Orphanet for rare diseases), and prevalence for one country or global average prevalence for all countries was used as an estimate for other countries, taking into account ageing by using the evolution of the population structure (2012–2016) derived from the 2012 ageing report (5).

### Step 5: Determination of uncertainty surrounding the estimations

The variability surrounding estimations was addressed in two ways: a deterministic one-way sensitivity analysis to identify drivers of the model outputs and a probabilistic sensitivity analysis to quantify uncertainty around the model outputs. The following parameters were included in the sensitivity analysis: the time to market after marketing authorization, time to reach peak of sales for new drugs (treating rare or non-rare diseases), price reduction of generic or biosimilar drugs, generic and biosimilar penetration, time to reach maximum of generic and biosimilar penetration, impact of generic and biosimilar drug entry on branded drug price, and reimbursement rate. Moreover, probabilistic sensitivity analysis specifically addressed the uncertainty surrounding the experts’ inputs related to the budget impact analysis of the new drugs.

Additionally, in order to account for the uncertainty regarding the reimbursement rate applied for all medicine, including in hospitals, a specific scenario was developed in which the reimbursement rate was accounted only in the outpatient sector.

Deterministic sensitivity analyses involve running the model for different values of model inputs, which are specified by the user, and comparing the obtained results. For this model, all parameters were increased and decreased by 30% of the original value, in order not to overweigh a parameter's variation compared to the others. This allowed us to see which parameter was a key driver of the model. The results of the deterministic one-way sensitivity analysis on the pharmaceutical net budget impact were presented per country as tornado diagrams, representing the range of variation of the pharmaceutical net budget impact for alternative values of several parameters.

With probabilistic sensitivity requiring specifying statistical distributions for all influential parameters surrounded with uncertainty, the model has to be run a large number of times (for different sets of input values drawn at random from pre-specified statistical distributions), providing statistical distributions for model results. In this model, a uniform distribution was used for each parameter of the model. This allowed for each value between the bounds of the range to have the same probability to be generated, meaning that uncertainty was reflected without any (*a priori*) assumptions. For some values, the bound obtained was not realistic (e.g., higher than 100 or lower than 0 for a percentage, or lower than 0 for a positive value), in which case coherent bounds were used. The model was subsequently run 1,000 times, the aggregated results of each simulation for each country and each perspective were saved and plotted, and basic statistics were computed (mean, standard error, median, minimum, maximum, and first [25%] and third [75%] quartiles).

Results of the probabilistic sensitivity analysis were presented as:

1. Probability curves showing the probability to obtain a budget impact lower than or equal to a chosen value (cumulative distributions of simulated budget impacts).
2. Scatterplots showing the spread of the ‘savings’ related to generics and ‘additional costs’ related to new branded medicinal products from simulations.

## Results^2^

### Budget impact analysis (base case)

Budget impact analysis showed that during the period of interest, all countries would experience a drug budget reduction, except Poland, which would experience an increase of +€41 million. Decrease in drug expenditure was the highest for the UK with −€9,367 million, followed by France with −€5,589 million, and farther behind by Germany with −€831 million and Greece with −€808 million, Portugal with −€243 million, and finally Hungary with −€84 million (Table 1).

**Table 1:** Net pharmaceutical budget impact during 2012–2016 per country from the healthcare public payer perspective (millions €)

Country	Net pharmaceutical budget impact during 2012–2016 (millions €)
France	−5,589
Germany	−831
Greece	−808
Hungary	−84
Poland	+41
Portugal	−243
UK	−9,367

**Table 2:** Reimbursement rate applied only for the retail chain for all countries versus the base case (net budget impact 2012–2016 (2011 €) for retail and hospital pharmaceutical expenditure from the healthcare public payer perspective (millions €))

Scenario number		Base case	10
	France	−5,589	−5,727
	Germany	−831	−827
Country	Greece	−808	−808
	Hungary	−84	−79
	Poland	41	26
	Portugal	−243	−277
	UK	−9,367	−9,367

Forecasting results are further described and discussed in a separate article, ‘EU pharmaceutical expenditure forecast (15)’.

### Deterministic one-way sensitivity analysis

**Fig. 4:**
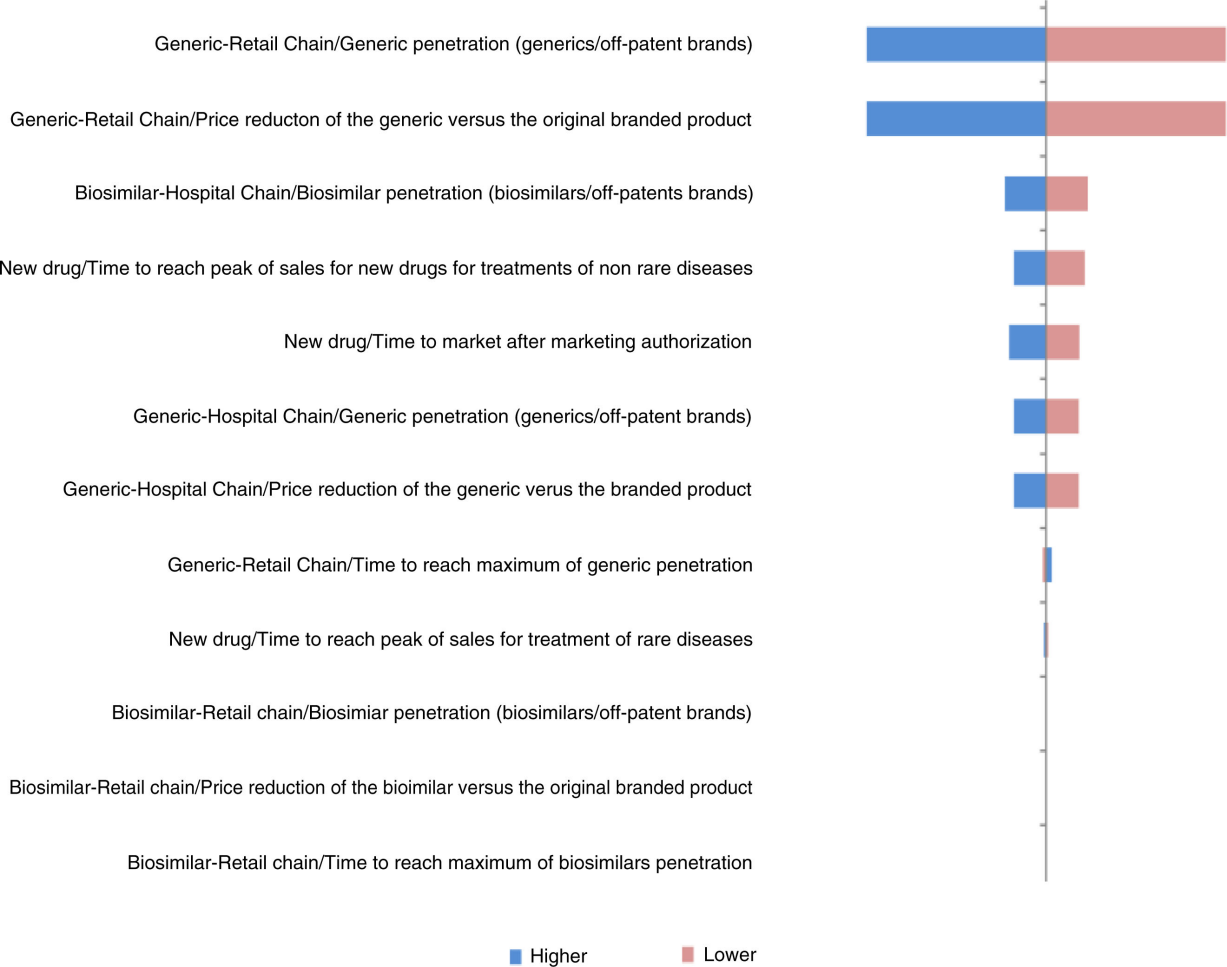
Change in pharmaceutical budget impact from the healthcare public payer perspective (millions €). Tornado diagram (with all parameters increased and decreased by 30%) for the UK.

**Fig. 5:**
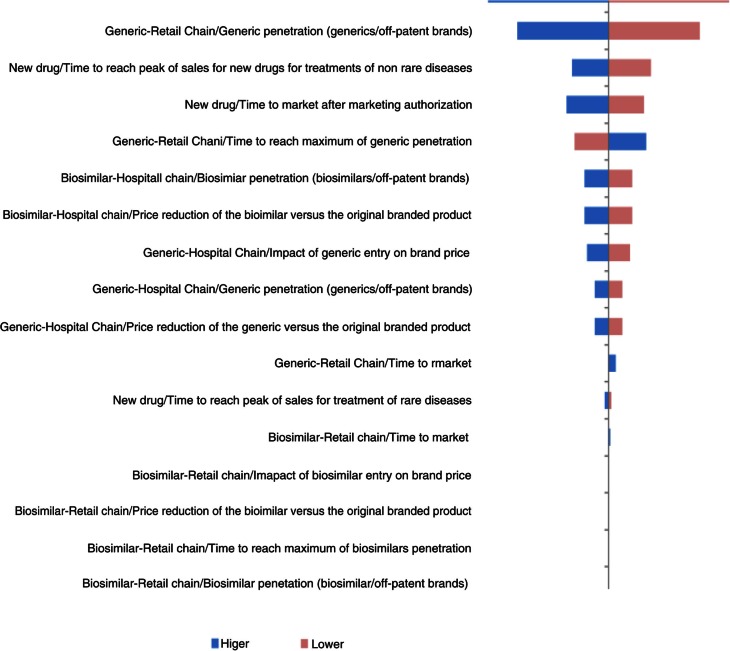
Change in pharmaceutical budget impact from the healthcare public payer perspective (millions €). Tornado diagram (with all parameters increased and decreased by 30%) for France.

**Fig. 6:**
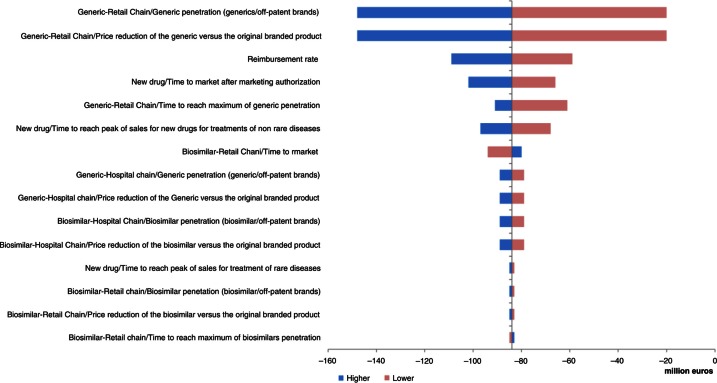
Change in pharmaceutical budget impact from the healthcare public payer perspective (millions €). Tornado diagram (with all parameters increased and decreased by 30%) for Hungary.

Three parameters had important impacts in the UK, France, and Hungary: price reduction of generics versus original branded drugs, penetration rate of generics via retail chain distribution, and reimbursement rate (Figs. 4–6). Other parameters of note for the UK were penetration rate of biosimilars and price reduction of biosimilars versus original branded drugs via hospital chain distribution (Fig. 4). Influential parameters for France were time to reach peak of sales for new drugs used to treat non-rare diseases and time to market for the new drug after marketing approval (Fig. 5). This last parameter was also a determining factor for Hungary (Fig. 6).

**Fig. 7:**
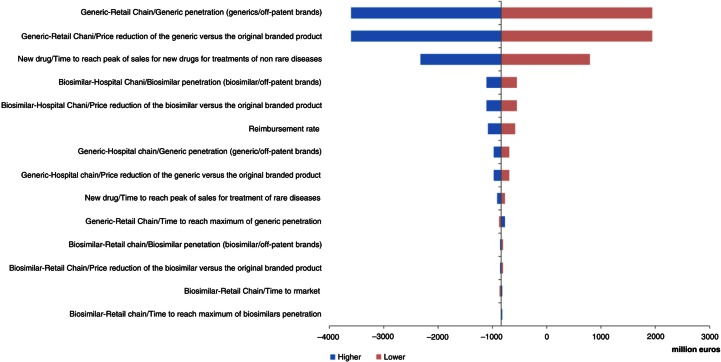
Change in pharmaceutical budget impact from the healthcare public payer perspective (millions €). Tornado diagram (with all parameters increased and decreased by 30%) for Germany.

In Germany, price reduction of generics versus original branded drugs and penetration rates of generics via retail chain distribution were the main impacting factors. The time to reach peak of sales for new drugs used to treat non-rare diseases was also an essential parameter (Fig. 7).

**Fig. 8:**
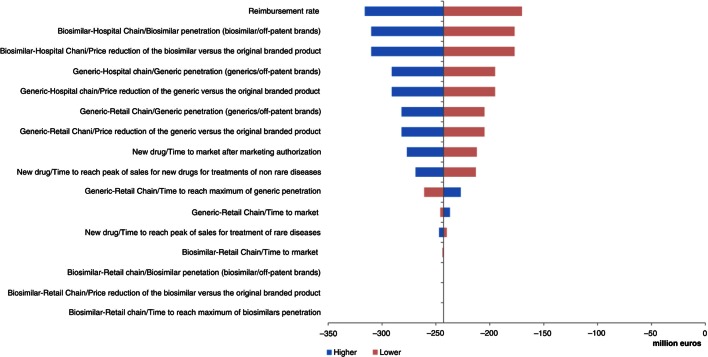
Change in pharmaceutical budget impact from the healthcare public payer perspective (millions €). Tornado diagram (with all parameters increased and decreased by 30%) for Portugal.

**Fig. 9:**
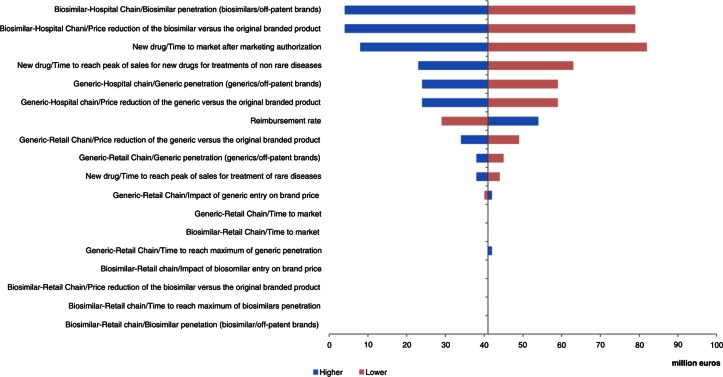
Change in pharmaceutical budget impact from the healthcare public payer perspective (millions €). Tornado diagram (with all parameters increased and decreased by 30%) for Poland.

In Portugal and Poland, the model was much more sensitive to variations in input parameters (Figs. 8 and 9). Key drivers of the model outputs were seen on the hospital distribution chain parameters, first on the price reduction and penetration rate of biosimilars, and then on the price reduction and penetration rate of generics.

**Fig. 10:**
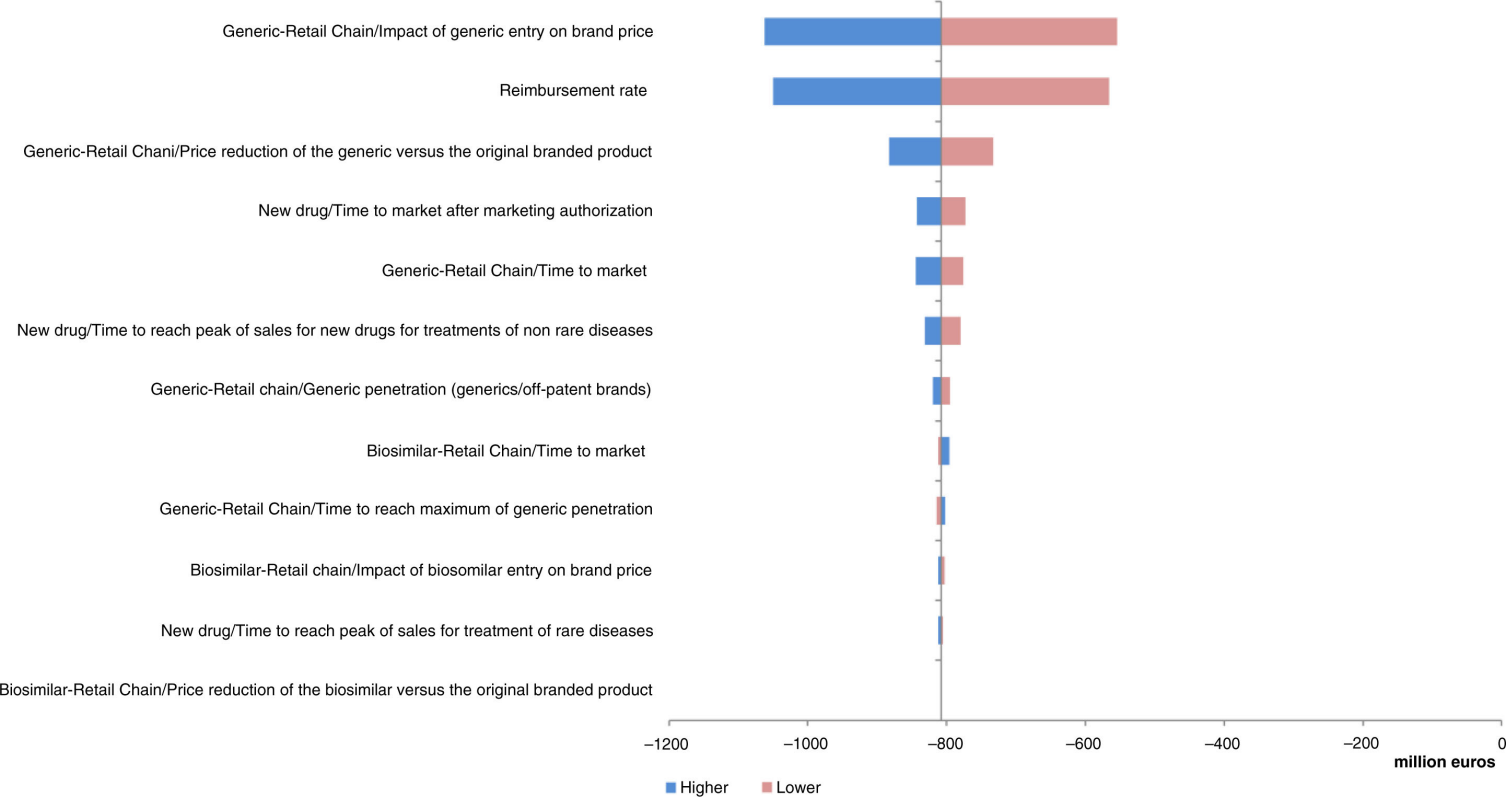
Change in pharmaceutical budget impact from the healthcare public payer perspective (millions €). Tornado diagram (with all parameters increased and decreased by 30%) for Greece.

The most important parameters influencing the model, in Greece, were the impact of entry of the generics on the original branded drug price and the reimbursement rate, followed by the price reduction of generics versus original branded drugs through retail chain distribution (Fig. 10).

The analysis showed that, in all countries except Portugal and Poland, two parameters had the greatest influence on the results of the model: the price reduction of generics versus original branded drugs and penetration rate of generics via the retail chain distribution.

Reimbursement rate applied only for the retail chain for all countries.

Applying the reimbursement rate only to the retail chain had a small impact on the pharmaceutical budget of each country (Table 2).

### Probabilistic sensitivity analysis

**Fig. 11:**
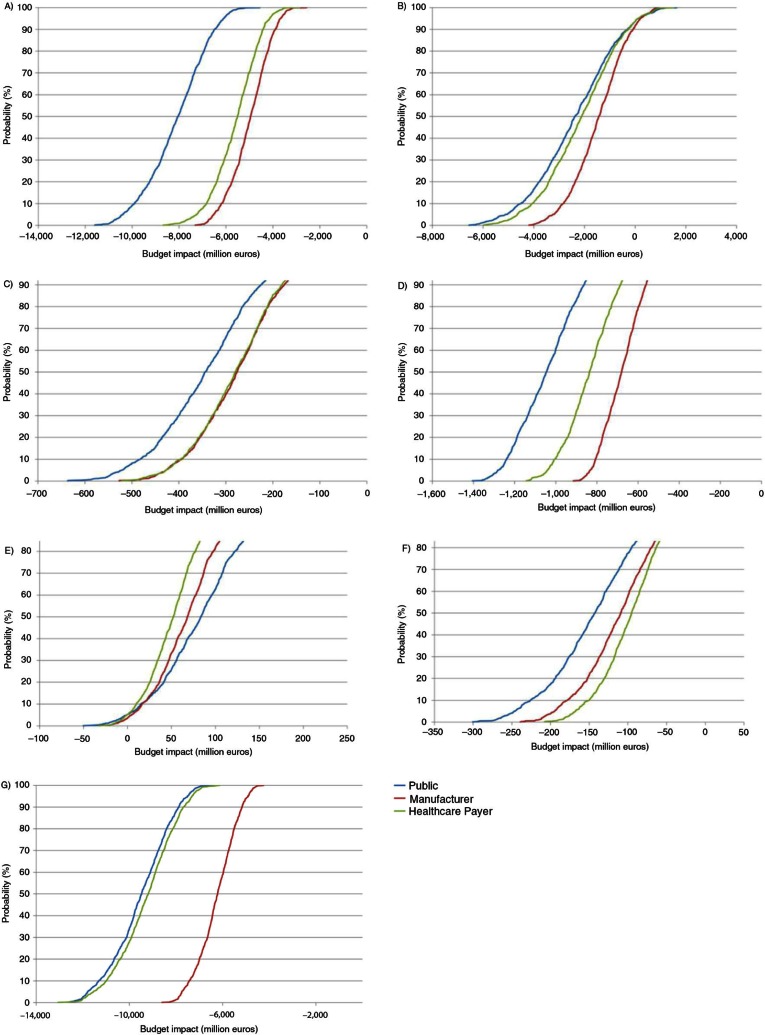
Probabilistic sensitivity analysis: budget impact probability curve for (A) France, (B) Germany, (C) Portugal, (D) Greece, (E) Poland, (F) Hungary, and (G) the UK.

**Fig. 12:**
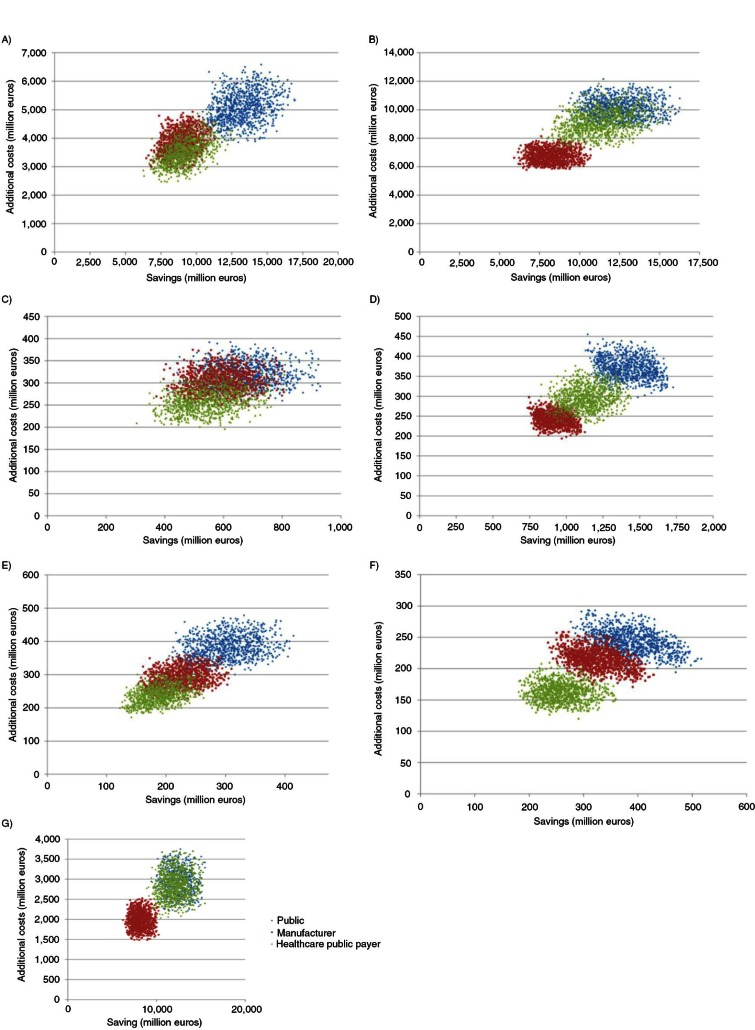
Probabilistic sensitivity analysis: scatterplot for (A) France, (B) Germany, (C) Portugal, (D) Greece, (E) Poland, (F) Hungary, and (G) the UK.

Cumulative distributions of simulated budget impacts are presented in Fig. 11, and scatterplots of the ‘savings’ according to the ‘additional costs’ are presented in Fig. 12.

The probabilistic sensitivity analysis allowed defining the probability of occurrence of a predetermined impact. This was illustrated in Fig. 11 with two countries with a large difference in terms of net pharmaceutical budget impact during 2012–2016 from the healthcare public payer perspective, the UK (−€9,367 million) and Portugal (−€243 million). With quite large uniform laws, half of the simulations were between −€10,133 million and −€8,359 million for the UK (Δ = 21%) and between −€341 million and −€224 million for Portugal (Δ = 52%) from a healthcare public payer perspective.

In Germany, the probabilistic sensitivity analysis showed a very low net budget impact compared to the base case. Half of the simulations were between −€3,203 and −€1,116 million with a base case at −€831 million. This was explained by the fact that the time to market for a new drug after marketing approval was fixed to zero in the base case, so the only possible evolution of this parameter in the sensitivity analyses was an increased time. This provided an asymmetric variation in one single direction, as time to market could not be negative. Moreover, time to market for the new drug was found to be a key driver of the results.

## Discussion

This study provided a new, highly flexible model that allows testing robustness and a new methodology that freed forecasting from the historical data, which were the main basis in most approaches.

The new methodology was used in this study to forecast pharmaceutical expenditure in seven EU MS over a 5-year forecasted period. Results of this forecasted project showed a consistent, but variable in magnitude, reduction in pharmaceutical expenditure in all countries with the exception of Poland. Two parameters had a great influence on expenditure in the selected countries, apart from Portugal and Poland: price reduction of generics versus original branded drugs and penetration rate of generics via retail distribution chain. These parameters were identified using a deterministic one-way sensitivity analysis, which permitted the identification of the main parameters impacting the pharmaceutical expenditure forecast of each country. This methodology measured the uncertainty surrounding the model's results and showed that the projected net budget impact was not altered with variations in parameters. Different pricing and reimbursement policy decisions on health expenditures were also tested with this methodology, providing probabilistic analysis to support policy decision making.

This modelling exercise was based on several assumptions discussed below.

For the generic model, hospitals were assumed to exclusively use generic or biosimilar products, when available. However, this is not always the case in the real world, as hospitals can use branded drugs, although only if their price is lower than or equal to the generic or biosimilar product's price. Thus, with this premise, this hypothesis had no consequence on the reliability of the model outputs.

The risk of drug development failure was estimated by therapeutic area, due to differences related to new drugs’ features which depend on the therapeutic area. This relevant methodology fits the budget impact analysis that was also oriented per therapeutic area.

The budget impacts of drugs approved in 2010 and 2011 were estimated by the board of experts according to the sales already generated and to the new medicine's ASMR assessment by the HAS complemented by the SMC review. Recommendations were assumed to be comparable in the seven countries even if it was not certain that this was the case. The HAS and SMC are the only health authorities that provided a comprehensive and argued review of all products reaching the market at the time surrounding launch. Both agencies use different methodologies: the SMC relies mainly on cost-effectiveness ratios, and the HAS relies mainly on clinical efficacy and effectiveness. Therefore, this method was considered to provide a reasonable view of the product's potential market value used for the forecasting exercise.

The reliability of the methodology used for assessing the budget impact of new oncology drugs in Greece, Hungary, Poland, and Portugal was confirmed using Germany instead of France as reference country, with stable results. Except for Poland, which seems to be enjoying a substantial increase in its gross domestic product, most countries are experiencing a tight overall budget and might have difficulties finding new revenue to fund additional expensive treatment options. Therefore, this method was considered to provide a reasonable estimate of oncology drugs’ impact in those countries.

New drugs to be approved, such as combinations of already approved products, me-too drugs, and new formulations of already approved products with no additional major clinical value, were considered to have no budget impact. However, having no budget impact does not mean not having any sales. In fact, nowadays, with increased pressure on EU MS budgets, only marginally innovative and non-innovative products are not expected to impact drug expenditure budgets. Such products will likely generate substantial sales but only by shifting budgets from older to new products. HTA regulations at the national level and health insurances have shown a substantial strengthening trend that is making it increasingly difficult for such products to impact the drug budget.

For the new drug model, peak sales were considered to be reached over a period of 3 years. This period is short compared to the average used in forecasting, where peak sales were usually achieved over 5 years. However, a faster uptake seemed appropriate as only innovative products were considered to affect the drug budget.

A ratio of ex-factory to retail sales was used as a rough aggregated estimate that was not representative of the detailed retail operating cost. In most countries, the cost of distribution chain is a complex calculation that depends on multiple parameters and should not be considered as a simple proportion. However, from an overall budget impact, it was an acceptable option to use such an overall ratio.

Non-orphan drugs with no phase II study results were considered unlikely to reach the market and generate significant sales before December 2016. However, even with negative phase II results, some pharmaceutical companies launch a phase III study in parallel with a new phase II study. Thus, some drugs not selected in the study might get access to the market during the study period. These cases were presumed to be quite rare, as risk of failure without a positive phase II remains high, and very few products are developed in parallel phases II and III.

Market development of products that were launched before 2010 and not expected to become generic in the considered period was not taken into account. Such products were likely to have some impact by increasing or decreasing their market share according to various possible events. This impact was expected to have low relevance to the study results due to the fact that it could lead at the same time to a quite similar increase and decrease of the pharmaceutical budget.

One global rate per country was applied for reimbursement on the total of pharmaceutical expenditures, including hospital expenditures, based on the mean of co-payment levels in place in each country. It was assumed, and confirmed in a specific scenario accounting for the reimbursement rate only in the outpatient sector, that this would not substantially affect the model's results.

Growth domestic product per capita was not considered to increase, even though it might have opened access to innovative molecules at a faster pace than it does today.

One limitation of this study is the amount of data needed to be collected and the amount of work incurred in order to analyze each drug individually. It has been especially complicated to obtain data on drugs’ patent expiry dates for all countries, due to the extensive range of patents available for one product (European and national patents). The large number of patent litigations surrounding the launch of generics and biosimilars is a consequence of the difficulty to appreciate the actual date of intellectual property (IP) loss, and the value of some additional patents used to reinforce IP protection. Several model inputs were based on board of experts’ insight (based on literature review data). The board looked at each new drug individually to assess its clinical potential and translate it into commercial potential. Such methods are simple, clear, and quite reliable when changes are occurring progressively. It allows for a case-by-case specific assessment but relies only on the experts’ capability and judgement. In this study, the board was supported by local experts providing reassurance on such limitations.

## Conclusion

Today, historical benchmarks are less reliable because of the disruption of the value appreciation of new drugs in most countries. This evidence-based methodology appears robust for pharmaceutical expenditure forecasting. It allows one to assess the uncertainty surrounding estimations, and it is independent from historical sales and market development, and thus particularly adapted to payers’ decisions making in a fast-changing environment. The reliability of this methodology could be confirmed by comparing the real pharmaceutical expenditure to the forecasted expenditure.

## Footnotes

1 Net budget impact is the difference between the additional costs of new drug entries and the savings associated with generic and biosimilar drug penetration.

## Footnotes

2 The full results related to the pharmaceutical expenditure forecast, along with a detailed description related to data collection and value assumptions for the model inputs, are presented in a separate article, ‘EU pharmaceutical expenditure forecast’ (15).
